# LARP7 is required for sex chromosome silencing during meiosis in mice

**DOI:** 10.1371/journal.pone.0314329

**Published:** 2024-12-05

**Authors:** Yukiko Tando, Atsuto Nonomura, Yumi Ito-Matsuoka, Asuka Takehara, Daiji Okamura, Yohei Hayashi, Yasuhisa Matsui

**Affiliations:** 1 Cell Resource Center for Biomedical Research, Institute of Development, Aging and Cancer, Tohoku University, Sendai, Japan; 2 Graduate School of Life Sciences, Tohoku University, Sendai, Japan; 3 Graduate School of Medicine, Tohoku University, Sendai, Japan; 4 School of Medicine Tohoku University, Sendai, Japan; 5 Department of Advanced Bioscience, Faculty of Agriculture, Kindai University, Nara, Japan; China University of Science and Technology, CHINA

## Abstract

Meiotic sex chromosome inactivation (MSCI) is an essential event in meiotic progression in mammalian spermatogenesis. We found that La Ribonucleoprotein 7 (LARP7) is involved in MSCI. LARP7 plays a role in fetal germ cells to promote their proliferation, but is once abolished in postnatal gonocytes and re-expressed in spermatocytes at the onset of meiosis. In spermatocytes, LARP7 localizes to the XY body, a compartmentalized chromatin domain on sex chromosomes. In germline-specific *Larp7*-deficient mice, spermatogenesis is arrested in spermatocytes, and transcription of the genes on sex chromosomes remained active, which suggests failure of meiotic sex chromosome inactivation (MSCI). Furthermore, the XY body in spermatocytes lacking *Larp7* shows accumulation of H4K12ac and elimination of H3K9me2, suggesting defective chromatin silencing by abnormal epigenetic controls. These results indicate a new functional role for LARP7 in MSCI.

## Introduction

In meiosis, homologous chromosomes undergo synapsis and recombination to promote genetic diversity in offspring. Meanwhile, the sex chromosomes, X and Y chromosomes, only partially undergo synapsis via their pseudo-autosomal regions. The remaining unsynapsed regions of the sex chromosomes are heterochromatinized after the late zygotene stage in meiosis, which are then compartmentalized and form an XY body, and the sex chromosomes are transcriptionally silenced in a process known as meiotic sex chromosome inactivation (MSCI) [1, 2]. Mechanistically, MSCI is directed by DNA damage response (DDR) pathways, and is initiated by phosphorylation of a histone variant H2AX at serine 139 (γH2AX) by a serine/threonine-protein kinase, ataxia telangiectasia and Rad3-related (ATR), and Breast Cancer gene 1 C-Terminal (BRCT) domain–containing protein TOPBP1, an ATR activator [2–6]. γH2AX then spreads into sex chromosomes by the function of mediator of DNA damage checkpoint 1 (MDC1), a γH2AX-binding partner [7]. Following the initiation of MSCI, the repressive epigenetic modifications such as di- and tri-methylated histone H3 lysine 9 (H3K9me2 and H3K9me3) are dynamically controlled including their transient loss at mid-pachytene followed by reappearance by late diplotene [11], and heterochromatin protein1 β (HP1 β) and HP1γ are localized on the XY body during pachytene stage [8–10]. In contrast, other repressive marks, such as mono-, di- and tri- H3K27 methylation and trimethylation of H4K20, are largely excluded from the XY body during the pachytene and diplotene stages [11]. Lack of factors required for MSCI resulted in arrest of meiosis. For instance, germ cell specific conditional deletion of *Topbp1* or *Setdb1* encoding a histone H3-lysine-9 methyltransferase, or systematic deletion of *Mdc1* causes germ cell elimination associated with defective MSCI at pachytene spermatocyte [6, 7, 10, 12, 13].

LARP7, a member of the La and La-related protein (Larp) family is known as a component of the 7SK snRNP (small nuclear ribonucleoprotein) and regulates transcription elongation by RNA polymerase II (Pol II) [14, 15]. Transcription elongation is stimulated by the positive transcription elongation factor b (P-TEFb), and 7SK snRNP forms a complex with P-TEFb to suppress its activity. If any components of the 7SK snRNP including Larp7 are decreased, the complex becomes destabilized, and P-TEFb is released as an active form [14, 15]. Previously, we demonstrated that LARP7 stimulates proliferation of germ cells in mouse embryos via transcriptional repression of a cyclin-dependent kinase inhibitor (CDKI) gene [16]. Because *Larp7*-null mice die between embryonic day 17.5 (E17.5) and birth, we could not determine a role of LARP7 in postnatal germ cells. More recently, a study demonstrated that a germline-specific *Larp7* depletion using the *Stra8-Cre* driver results in arrest of spermatogenesis during meiosis and spermiogenesis, and remaining spermatozoa are nonfunctional [17]. This study also indicated that LARP7 is involved in splicing regulation via 2’-O-methyaltion of U6 snRNA modification by box C/D snoRNA protein complex (snoRNP) in spermatogenic cells, and LARP7 co-localizes with fibrillarin (FBL), a component of box C/D snoRNP in nucleus [18] in the spermatocytes [17]. During our attempt to examine a role of LARP7 in postnatal germ cells, we found that LARP7 not only co-localizes with FBL in nucleolus, but also in the XY body in spermatocyte. This suggests that LARP7 has a function in the XY body in addition to its function in the nucleolus. Therefore, we aimed to elucidate a possible function of LARP7 in the XY body by using germline-specific *Larp7*-deficient mice with *Vasa-Cre* driver, which enable to earlier onset of *Larp7* deletion during spermatogenesis compared with those with *Stra8-Cre*, and examined their phenotype in spermatogenesis. As a result, meiosis was arrested in testes of *Larp7* cKO mice, leading to a failure in sperm formation as in the previous study. Furthermore, we newly found that these spermatocytes exhibited impaired MSCI, with aberrant histone modifications in the XY body. From these results, we postulate that LARP7 is involved in the establishment of repressive histone modification status in the XY body, which contribute to MSCI.

## Materials and methods

### Mice

*Larp7*^f/f^ mice was generated by the RIKEN CDB, Japan. Briefly, mouse genomic fragments containing homology arms and a conditional knockout (cKO) region were amplified from BAC clone by (Polymerase Chain Reaction) PCR, and were sequentially assembled into a targeting vector together with recombination sites and selection markers. The linearized vector [19] was subsequently delivered to ES cells (TT2 cells [20]) via electroporation, followed by drug selection against G418, PCR screening, and southern blot analysis. Targeted ES cells are injected into C57BL/6J 8-cell stage embryos to generate chimeric mice. To obtain germline-specific knockout mice, we crossed *Larp7*^f/f^ mice to *Vasa-Cre* mice [21]. The mice were maintained and bred in an environmentally controlled and specific-pathogen-free facility, the Animal Unit of the Institute of Development, Aging and Cancer (Tohoku University), according to the guidelines for experimental animals defined by the facility. Animal protocols were reviewed and approved by the Tohoku University Animal Studies Committee. Mice were sacrificed by cervical dislocation, Primers used for genotyping are listed in S1 Table.

### Quantitative RT-PCR

Total RNA was extracted from cells using a RNeasy Micro Kit (Qiagen 74004) according to the manufacturer’s instructions. RNAs were reverse-transcribed using SuperScript III reverse transcriptase (Invitrogen 18080093) and the random primers (Promega C1181). Real-time PCR was performed using Power SYBR Green PCR Master Mix (Applied Biosystems 4367659). Thermal conditions were 2 min at 50°C, 10 min at 95°C, and 45 cycles of 15 sec at 95°C and 60 sec at 60°C. Sequences of the primers used for the PCR reaction are shown in S1 Table. The *Arbp* transcript was used as an internal control. The relative expression was analyzed using comparative CT method.

### Histological examination

Testes and epididymis were fixed overnight at 4°C in Bouin’s solution with rotation, and then were embedded in paraffin. Five-micrometer-thick serial sections were cut and mounted on 3-aminopropyltriethoxysilane (APS) -coated slides (Matsunami APS-01); the mounted sections were deparaffinized, stained with hematoxylin and eosin, and examined using an optical microscope (Leica). Digital images were obtained using a LAS4.4 (Leica).

### Immunostaining

Tissues were fixed in 4% paraformaldehyde for overnight at 4°C and embedded in paraffin and sectioned into 5 μm. After deparaffifnization, antigen retrieval was performed in citrate buffer, pH 6.0 (10mM sodium citrate), and the sections were blocked by 5% BSA / 0.1% Triton X-100 in Phosphate Buffered Saline (PBST), incubated with the first antibodies for overnight at 4°C, and then incubated with the secondary antibodies and 1 μg/mL DAPI for 1 h at room temperature. Primary antibodies were: anti- LARP7 (abcam ab105682, 1:200), anti-SCP3 (abcam ab97672, 1:500), anti-SCP3 (abcam ab15093, 1:500), anti-γH2AX (upstate 05–636, 1:500), anti-H1t (kindly provided by Dr. Mary Ann Handel, 1:1000) [22], anti-FBL (Neuromics CH22129, 1:2000), anti-phosphorylated RNA polymerase II (abcam ab5095, 1:2000), anti-H3K4me2 (upstate 07–030, 1:500), anti-H3K4me3 (abcam ab8580, 1:500), anti-H3K9me2 (upstate 07–441, 1:500), anti-H3K9me3 (abcam ab8898, 1:500), anti-H3K27me3 (upstate 07–449, 1:500), anti-H4K9ac (abcam ab10812, 1:700), anti-H4K12ac (abcam ab46983, 1:500), anti-H4K16ac (abcam ab109463, 1:200), anti-MDC1 (proteintech 24721-1-AP). The sections were washed by PBST after primary and secondary antibody treatments. Samples were mounted using Vectashield (Vector H-1000) and observed under a TCS SP8 confocal laser scanning microscope (Leica). The fluorescence intensity of the XY body or outside of the XY body was measured in 3~5 cells from each of at least five testicular tubules in each individual using the histogram option of LAS X software (Leica), and the average intensity for somatic cells was then calculated. Relative signal intensity in the XY body or outside the XY body were standardized by the average values of the fluorescence intensity of 15 surrounding somatic cells in each individual.

### Chromosome spread sample preparation

Chromosome spread sample were prepared according to previous report (Methods Mol Biol 2018:1861:113–129). Briefly, after removing the tunica albuginea, the testes were loosened in a well with Phosphate Buffered Saline (PBS) on ice. The seminiferous tubules were transferred to wells containing Hypobuffer (600 mM Tris HCl (pH 8.2), 500 mM sucrose, 170 mM natriumcitraat (pH 8.2), 500 mM ethylenediaminetetraacetic acid (EDTA), 100 mM dithiothreitol (DTT), and proteinase inhibitors (Rhoche 4693116001), and were spread with tweezers to expose the seminiferous tubules to the buffer. The seminiferous tubules were incubated on ice for 1.2 to 2 hours. The four to six tubules were then transferred to a glass slide with a drop of 30 μl of ice-cold 100 mM sucrose with tweezers, and crushed with the tips of two tweezers. 30 μl of 100 mM sucrose was added to the suspension, and half of it was applied to glass slides immersed in fixing solution (2% Paraformaldehyde, 10 mM sodium borate buffer (pH9.2), 0.02% sodium dodecyl sulfate (SDS), 0.1% Triton X-100), were spread and dried overnight. The slides were then washed twice with 0.4% Photo-Flo (Kodak 146–4510) for 2 minutes, dried, and stored at -80°C.

### Fluorescence in situ hybridization (FISH) and immunostaining

After placing the glass slides with the chromosome spread samples on a 70°C hot plate for at least 30 min, the spread specimen on slides were incubated with a drop of 0.002% pepsin/0.1 N HCl for 1 min at room temperature, rinsed twice with distilled water, and dried at 70°C on a hot plate. Mouse Chromosome XY probe (Chromosome science lab MXY-10) were applied to cell specimens, covered with cover glass, denatured at 70°C on a hot plate for 5 min, and hybridized overnight at 37°C. The slides were immersed in 50% formamide/2xSSC at 37°C for 20 min, rinsed with 1xSSC, immersed in 1xSSC for 15 min, incubated in blocking buffer at room temperature for 1 hr. The specimen were then reacted with anti-γH2AX antibody overnight at 4°C, with secondary antibody and 1μg/ml DAPI for 1 hour at room temperature, and then sealed with Vectashield.

### Western blotting

Cells were suspended in Lysis buffer (50 mM Tris-HCl pH7.4, 150 mM NaCl, 1% TritonX-100, 5 mM EDTA, protease inhibitors), and sonicated by Bioruptor (Diagnode) for 30 sec ON with 30 sec interval. The cell lysates were separated on SDS-PAGE gels and transferred to PVDF membrane (Bio Rad 1620177). The membrane was blocked with 5% bovine serum albumin (BSA) in PBST, and then sequentially incubated with primary antibodies for LARP7 (Bethyl A303-723A, 1:1000) and β-Actin (abcam ab6276, 1:5000), and horseradish peroxidase-conjugated secondary antibodies (Jackson 115-035-062, 111-035-144). The blots were analyzed within Amersham ImageQuant 800 after treated with enhanced chemiluminescence substrate (Clarity Western ECL Substrate, BioRad 1705060).

### Immunoprecipitation

Tissues were lysed with buffer A (10 mM HEPES pH7.9, 10 mM KCl, 1.5 mM MgCl2, 0.05% NP40 and 0.5 mM DTT) on ice for 10 min, centrifuged at 3000 rpm for 5 min, and the precipitated nuclear pellets were further lysed in high salt buffer B (20 mM HEPES pH7.9, 450 mM NaCl, 25% Glycerol, 0.2 mM EDTA, 0.5 mM DTT, 1% NP40 and 0.5% SDS) for 20 min. The nuclear extract was incubated with primary antibodies for LARP7 (Bethyl A303-723A), γH2AX (abcam ab81299), or normal rabbit IgG (CST 2729S) overnight at 4°C and then pulled down with protein G Dynabeads (Thermo Fisher, 10004D), or directly pulled down by antibody-conjugated protein G Dynabeads (Thermo Fisher, 10004D) at 4°C for overnight. After washing with 0.15 M NaCl buffer (50mM HEPES pH7.5, 0.15M NaCl, 0.1% NP-40) once, and 0.3 M NaCl buffer (50mM HEPES pH7.5, 0.3M NaCl, 0.1% NP-40) twice, beads-bound protein complexes were solubilized by boiling in 4x Laemmli buffer (Bio Rad 1610747) and detected with the antibodies for LARP7 (Bethyl A303-723A, 1:1000) or γH2AX (abcam ab81299, 1:5000).

### Isolation of spermatogenic cells from mouse testes

Isolation of spermatogenic cells from testes was performed according to the previous reports [23, 24]. After the tunica albuginea was removed, testes were incubated at 32°C for 25 min in 6 mL of Gey’s Balanced Salt Solution (GBSS; Sigma-Aldrich G9779) containing 1.2 mg/mL of Collagenase Type I (Sigma-Aldrich C0130), and the seminiferous tubules were dissociated. Interstitial cells were removed by filtration with the Cell strainer (Falcon 352340). Seminiferous tubules retained on the filter were collected and incubated at 32°C for 25 min in Gey’s Balanced Salt Solution (GBSS, Sigma 9779-500ML) containing 1.2 mg/mL of Collagenase Type I and 5 μg/mL DNase (Roche 11284932). Cell aggregates were sheared gently by 10 rounds of pipetting with a wide orifice plastic transfer pipet and filtered through the Cell strainer to remove cell clumps. Cells were washed with GBSS and then resuspended in GBSS containing 1% fetal bovine serum (FBS). Twenty million cells were diluted in 2 mL of GBSS containing 1% FBS and stained with 5 μg/mL of Hoechst 33342 (Invitrogen H3570) for 1 h at 32°C. Cells were kept on ice and protected from light until sorting. Before sorting, 0.25 μg/mL of propidium iodide (PI, BD 51-66211E) was added to the stained cells, and the mixture was filtered through the Cell strainer. The cells were sorted using a Becton- Dickinson FACS Aria II cell sorter. PI-positive cells reflect dead cells, which were first eliminated by gating, and PI-negative living cells were then further fractionated to spermatogonia, spermatocytes, and round spermatids according to their Hoechst staining patterns. Fluorescence of Hoechst 33342 has two emissions wave length (450nm, blue and 675 nm, red), and the stained cells can be separated by signal intensity for the two wavelengths, which partly reflects DNA content of the cells [25]. Confirmation of the purity of the sorted germ cells was evaluated by the assessing the expression of stage-specific germ cell marker genes as follows: *Gfra1* for spermatogonia, *Scp3* for spermatocytes, and *Acrv1* for spermatids. We obtained four and six biological replicates for spermatocytes for transcriptome and RT-qPCR, respectively.

### RNA sequencing

RNA-seq libraries were prepared from total RNA purified from sorted germ cells from four WT and *Larp7* cKO mice at 7 weeks old as biological replicates with a RNeasy Micro Kit (QIAGEN 74004). The libraries constructed by NEBNext Ultra II RNA Library Prep Kit for Illumina (BioLabs E7770S) were clonally amplified on a flow cell (Illumina) and sequenced on HiSeq2500 (HiSeq Control Software v2.2.58, Illumina) with 51-mer single-end sequences. Image analysis and base calling were performed using Real-Time Analysis Software (v1.18.64, Illumina). For gene expression analysis, reads were mapped to the mouse genome (UCSC mm10 genome assembly and NCBI RefSeq database) using TopHat2 (v2.1.0) [26] and Bowtie2 (v2.2.6.0) [27]. FeatureCounts (packaged in Subread v1.5.0-p2) [28] was used to calculate count data from BAM files. After TMM normalization by edgeR (v3.13) [29] operated in R (v4.1.0), differentially expressed genes were extracted with log2-fold-change > 1 or < -1 and threshold as FDR < 0.05. TMM-normalized count data were obtained by utilizing Tag Count Comparison-Graphical User Interface (TCC-GUI) platform (https://infinityloop.shinyapps.io/TCC-GUI/) [30]. R was used to generate Heatmap. The Database for Annotation, Visualization, and Integrated Discovery (DAVID v6.8, https://david.ncifcrf.gov/, Classification stringency: medium) [31] was used for functional annotation. Statistically significant (p < 0.05) GO terms were filtered by BH-corrected p-values with cut off value of FDR < 0.05.

### Statistical analysis

The significance of difference was assessed by the unpaired two-sided Student’s t test. *P* value < 0.05 was judged to be a statistically significant difference.

## Results

### Expression and localization of LARP7 in the postnatal testis

**Fig 1:**
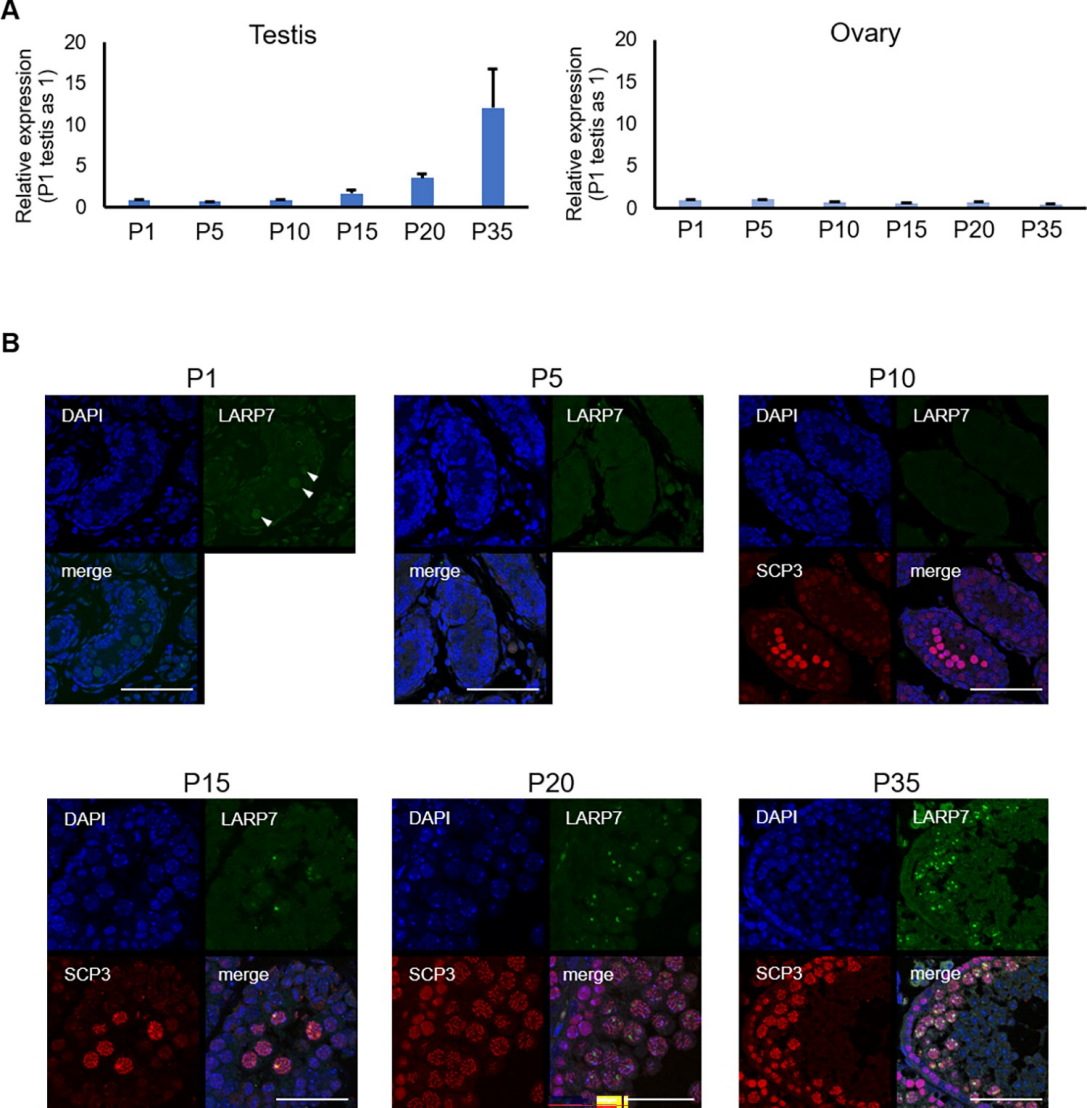
Expression of LARP7 in the germ cells at postnatal stages. A. Gene expression of *Larp7* in the testis and ovary after birth (Mean ± SE, n = 3). P: postnatal day. B. LARP7 (green) detected by immunostaining in the testis sections after birth. Arrowheads indicate germ cells. SCP3 (red) detected synaptonemal complex. Bars = 50 μm.

First, we examined the expression of Larp7 mRNA in the postnatal gonads, and the results show its progressive upregulation according to postnatal development in testis, with particularly marked increase after postnatal (P) 15 onwards (Fig 1A). In contrast, its expression was constantly low in ovary until P35, when most of oocytes are arrested in meiotic prophase, and it is consistent to a role of LARP7 in MSCI as described below. Because of the marked increase of *Larp7* expression in the postnatal testes, we examined the localization of LARP7 protein in postnatal testes by immuno-histological analysis (Fig 1B). At P1, LARP7 was diffusely localized in the germline nucleus. From P5 to P10, LARP7 signals once disappeared. After P15, multiple punctate signals appeared in the nucleus of spermatocytes, which are likely pachytene to diplotene stages (Fig 1B).

**Fig 2:**
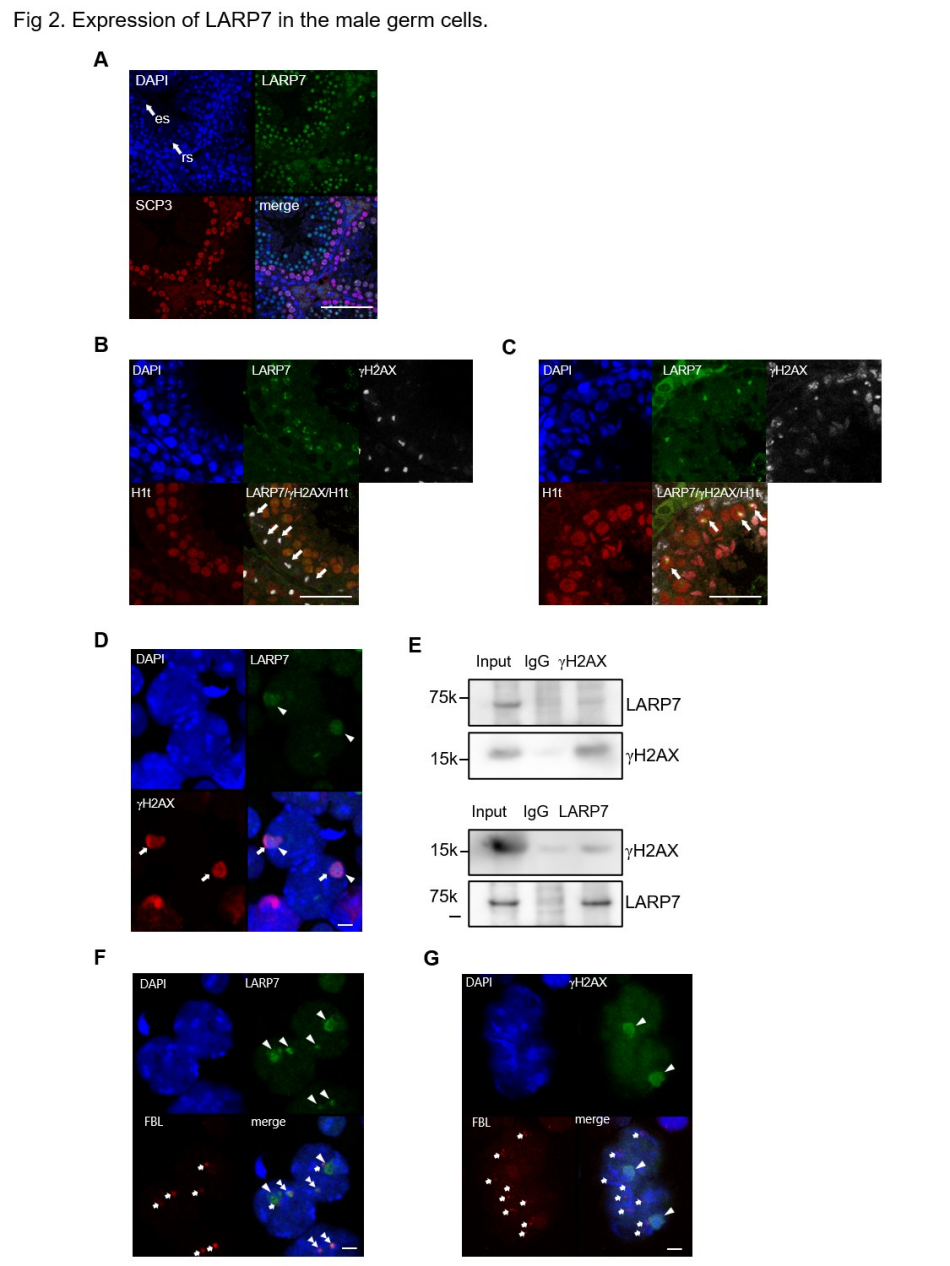
Expression of LARP7 in the male germ cells. A. LARP7 (green) and SCP3 (red) detected by immunostaining in the spermatocytes and spermatids of the testis sections from 5 weeks old mice. Arrows indicate round spermatid (rs) and elongated spermatid (es). Bar = 100 μm. B, C. LARP7 (green), γH2AX (white), and H1t (red) detected by immunostaining in the early stage (B) or mid-late stage (C) of pachytene spermatocytes of the testis section with nuclei counterstained by DAPI (blue) from 5 weeks old mice. Arrows indicate spermatocyte. Bars = 50 μm. D. LARP7 (green) and γH2AX (red) detected by immunostaining in the spermatocyte of chromosome spread sample with nuclei counterstained by DAPI (blue) from 5 weeks old mice. Arrowheads indicate LARP7 signal and arrows indicate γH2AX signal. Bar = 10 μm. E. Immunoprecipitation assay of LARP7 and γH2AX in mouse testes at P21. Anti-γH2AX or anti-LARP7 immunoprecipitated samples was blotted by anti LARP7 or γH2AX, respectively (lane 3). Testis lysate (Input, lane 1) and IgG IP (lane 2). Molecular weights are indicated on the left. F. LARP7 (green) and FBL (red) detected by immunostaining in the spermatocyte of chromosome spread sample with nuclei counterstained by DAPI (blue) from 5 weeks old mice. Arrowheads indicate LARP7 signal, arrows indicate FBL signal, and double arrowheads indicate colocalization of LARP7 and FBL. Bar = 10 μm. G. γH2AX (green) and FBL (red) detected by immunostaining in the spermatocyte of chromosome spread sample with nuclei counterstained by DAPI (blue) from 5 weeks old mice. Arrowheads indicate γH2AX signal and arrows indicate FBL signal. Bar = 10 μm.

In 5-week-old testis, signals of LARP7 were co-localized with γH2AX which is accumulated on sex chromosomes to forms the XY body [32, 33] in histone H1t-positive spermatocytes at mid-pachytene stage onwards [22], but the LARP7 signals were not observed in H1t-negative early pachytene spermatocytes (Fig 2B and 2C). The signals of LARP7 were also observed in the round spermatids, but not in the elongated spermatids in 5-week-old testis (Fig 2A). In spermatocytes, a signal of LARP7 in nuclei was co-localized with γH2AX which is accumulated on sex chromosomes to forms the XY body (Fig 2D). The interaction of LARP7 and γH2AX was confirmed by immunoprecipitation-western blot assay (Fig 2E).

Previous studies have reported that the nucleolar protein Fibrillarin (FBL) functions together with LARP7 in mouse spermatogenic cells [17]. Therefore, we closely examined the localization of FBL and LARP7. As the results in the previous report [17], FBL was co-localized only with a fraction of LARP7 in the nucleus of spermatocytes, while we found that FBL was rarely co-localized with a larger signal of LARP7 in the XY body (Fig 2F and 2G). These results suggest that LARP7 has a role in the XY body without interaction with FBL.

### Failure of spermatogenesis by deficiency of LARP7

**Fig 3:**
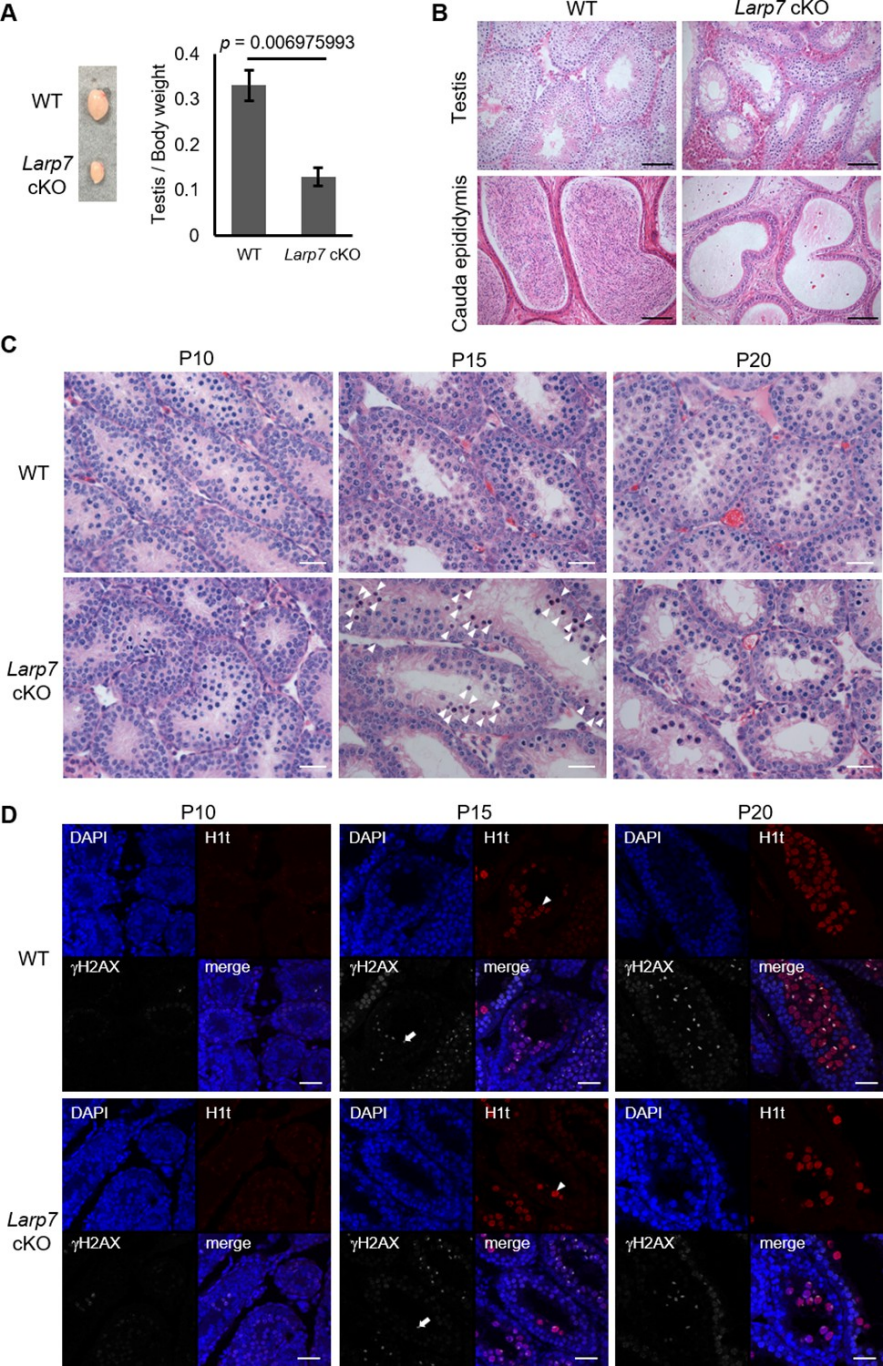
Abnormal spermatogenesis of *Larp7* cKO mice. A. Testes from *Larp7* cKO mice. Left, a representative image of testes from 5 weeks old mice; right, the average testis/body weight ratios from wild type and *Larp7* cKO mice (mean ± SE, n = 3). B. Representative hematoxylin and eosin (H&E) staining images of testis and epididymis from 8 weeks old wild type or *Larp7* cKO mice. Bars = 100 μm. C. Representative H&E staining images of testis sections from P10-P20 wild type and *Larp7* cKO mice. In *Larp7* cKO mice testis, meiosis was arrested at the spermatocyte stage (arrowheads in P15 indicate dead spermatocytes) and round spermatids was not detected at P20. Bars = 50 μm. D. Immunostaining of testis sections from P10-P20 wild type and *Larp7* cKO mice for H1t (red) and γH2AX (white) with nuclei counterstained by DAPI (blue). Arrowheads indicate H1t signal and arrows indicate γH2AX signal detected in P15. Both signals are detected in the same cell in each of wild type and *Larp7* cKO mice. Bars = 10 μm.

From the localized expression of LARP7 in the XY body in spermatocytes, we then investigated its possible novel functions in spermatocytes by generating its conditional knockout mice by crossing *Larp7* floxed mice with germ cell-specific *Vasa-Cre* mice [21] (S1A and S1B Fig). We confirmed significantly reduced Larp7 mRNA and its protein (S1C and S1D Fig), and significantly decreased nuclear LARP7 was also observed (S1E Fig). We found that the average weight of *Larp7* cKO testes was about 60% lower than that of controls (Fig 3A). Histological analysis of testis sections at P34 revealed that spermatogenesis in *Larp7* cKO testis was arrested at the spermatocyte stage, and there were no mature sperm in the epididymis (Fig 3B). The spermatogenic abnormality was first observed at P15 when spermatocytes first appeared (Fig 3C), which is concomitant with increased expression of LARP7 in spermatocytes in testis. In addition, H1t-positive mid- to late spermatocytes and round spermatids were observed after P15 in *Larp7* cKO testis, though their number was decreased compared with that in age-matched wild type testis (Fig 3D), suggesting meiotic arrest at late pachytene.

### Incomplete meiotic sex chromosome inactivation caused by deficiency of Larp7

**Fig 4:**
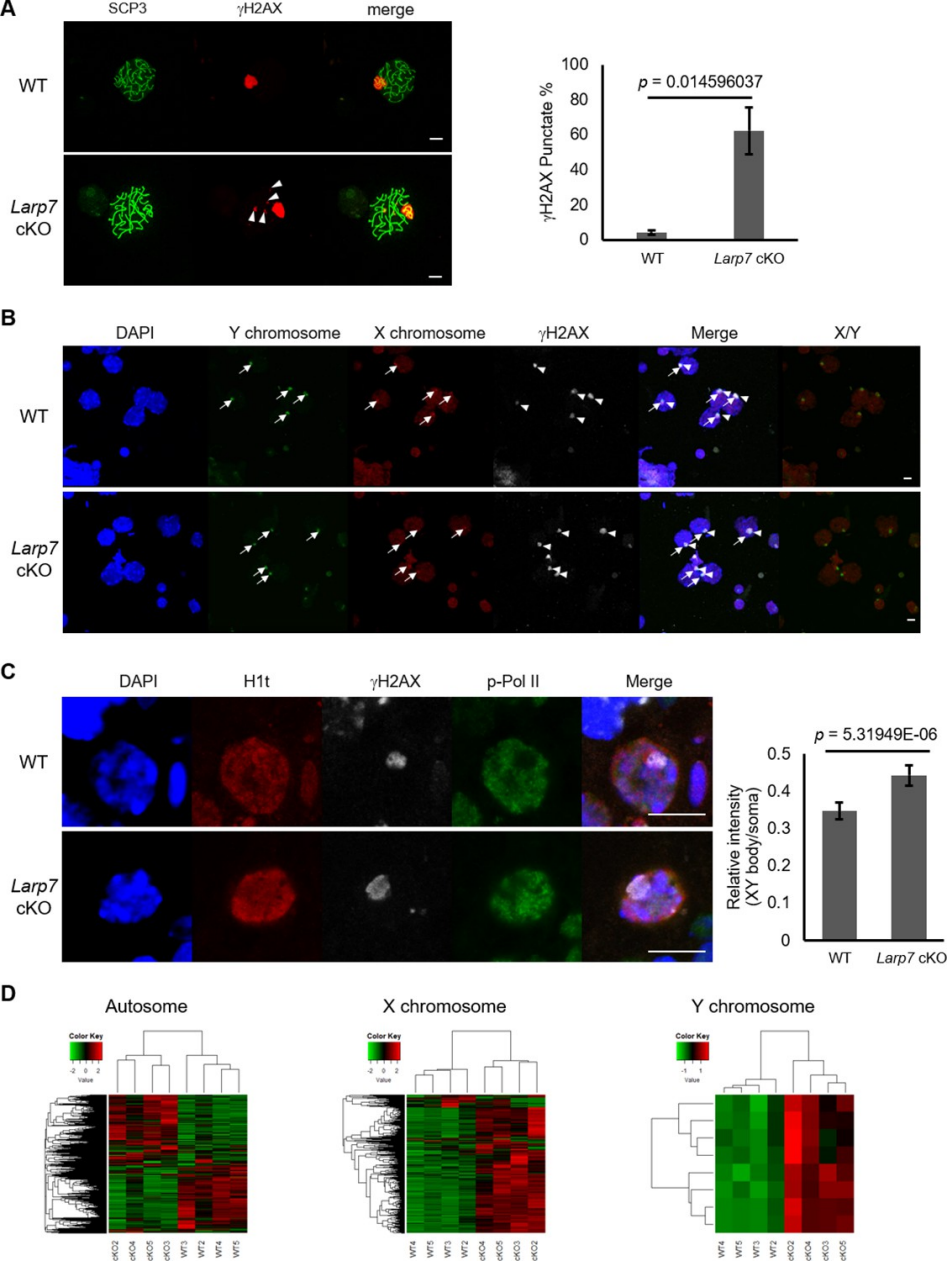
Incomplete MSCI in the *Larp7* cKO spermatocyte. A. Detection of SCP3 and γH2AX in wild type and *Larp7* cKO mice. Left, representative images of SCP3 (green) and γH2AX (red)) detected by immunostaining in the spermatocyte of chromosome spread sample of wild type and *Larp7* cKO mice at 5 weeks old. Right, quantification of punctate γH2AX signals outside the XY body in wild type and *Larp7* cKO spermatocytes (Mean ± SE, WT: n = 78 cells from 3 animals, *Larp7* cKO: n = 32 cells from 4 animals). Arrowheads indicate γH2AX signals outside the XY body. Bars = 10 μm. B. X (red) and Y (green) chromosome FISH combined with immunostaining with γH2AX (white) in the spermatocyte of chromosome spread samples of wild type and *Larp7* cKO mice at 5 weeks old. Arrows indicate X and Y chromosome, and arrowheads indicate the XY body. Bars = 10 μm. C. Detection of Phosphorylated RNA polymerase II (p-Pol II) in the spermatocyte of sections of wild type and *Larp7* cKO mice at 5 weeks old. Left, representative images of p-Pol II (green) and γH2AX (white) detected by immunostaining in the adult testis section of wild type and *Larp7* cKO mice. Right, quantification of relative intensity of p-Pol II in the XY body in H1t positive spermatocytes in wild type and *Larp7* cKO testes (Mean ± SE, n = 150 cells from 3 animals). Bars = 10 μm. D. Heatmap analysis of the expression of genes encoded in autosome, X chromosome, and Y chromosome in wild type and *Larp7* cKO spermatocytes at 7 weeks old (n = 4).

Since we found the co-localization of LARP7 and γH2AX in spermatocytes and their interaction, we predicted a role of LARP7 related to the XY body, and examined the localization of γH2AX in *Larp7* cKO testes. In addition to accumulation of γH2AX, which reflects the formation of the XY body, few additional small foci of γH2AX were sometimes observed in autosomes in the *Larp7* cKO spermatocytes, suggesting incomplete double-strand break repair (DSBR) in the *Larp7* cKO (Fig 4A). MDC1 was also localized in XY body in mid- to late-pachytene spermatocytes in the *Larp7* cKO testis as in wild type (S2 Fig). We further confirmed the formation of the XY body by using FISH for sex chromosomes, and observed co-localization of X and Y chromosomes with γH2AX in both wild type and *Larp7* cKO spermatocyte (Fig 4B). We then examined possible abnormalities of MSCI in H1t-posituve spermatocytes after mid-pachytene stage in *Larp7* cKO testis by quantification of phosphorylated RNA-polymerase II (p-Pol II) intensity representing transcriptional activity in the XY body. In the XY body in *Larp7* cKO spermatocytes, signals of p-Pol II were relatively higher compared with those in wild type (Fig 4C). We also compared the expression of sex chromosome genes in wild type and *Larp7* cKO spermatocyte by RNA-seq. As expected, the expression levels of most of genes on X and Y chromosomes in *Larp7* cKO spermatocytes were higher than those in wild type spermatocytes, while there are certain gene sets of upregulated or downregulated autosomal genes between WT and *Larp7* cKO spermatocytes (Fig 4D, S2 Table). Upregulation of representative sex chromosome genes (*Taf7l*, *Ccnb3*, *Ddx3y*, *Uba1y*) in *Larp7* cKO spermatocyte was confirmed by qRT-pCR (S3A Fig). The results suggest failed MSCI in the *Larp7* cKO spermatocyte, even though formation of XY body. Interestingly, functional annotation of downregulated genes in *Larp7* cKO spermatocytes demonstrated enrichment of spermatogenesis-related terms, in particular, those related to post-meiotic spermiogenesis such as cilium and sperm motility, which is related to the reduced spermiogenesis by meiotic arrest phenotype of *Larp7* cKO mice (S3 Fig and S4 Table).

### Changes of histone modifications in the XY body of Larp7 cKO spermatocyte

**Fig 5:**
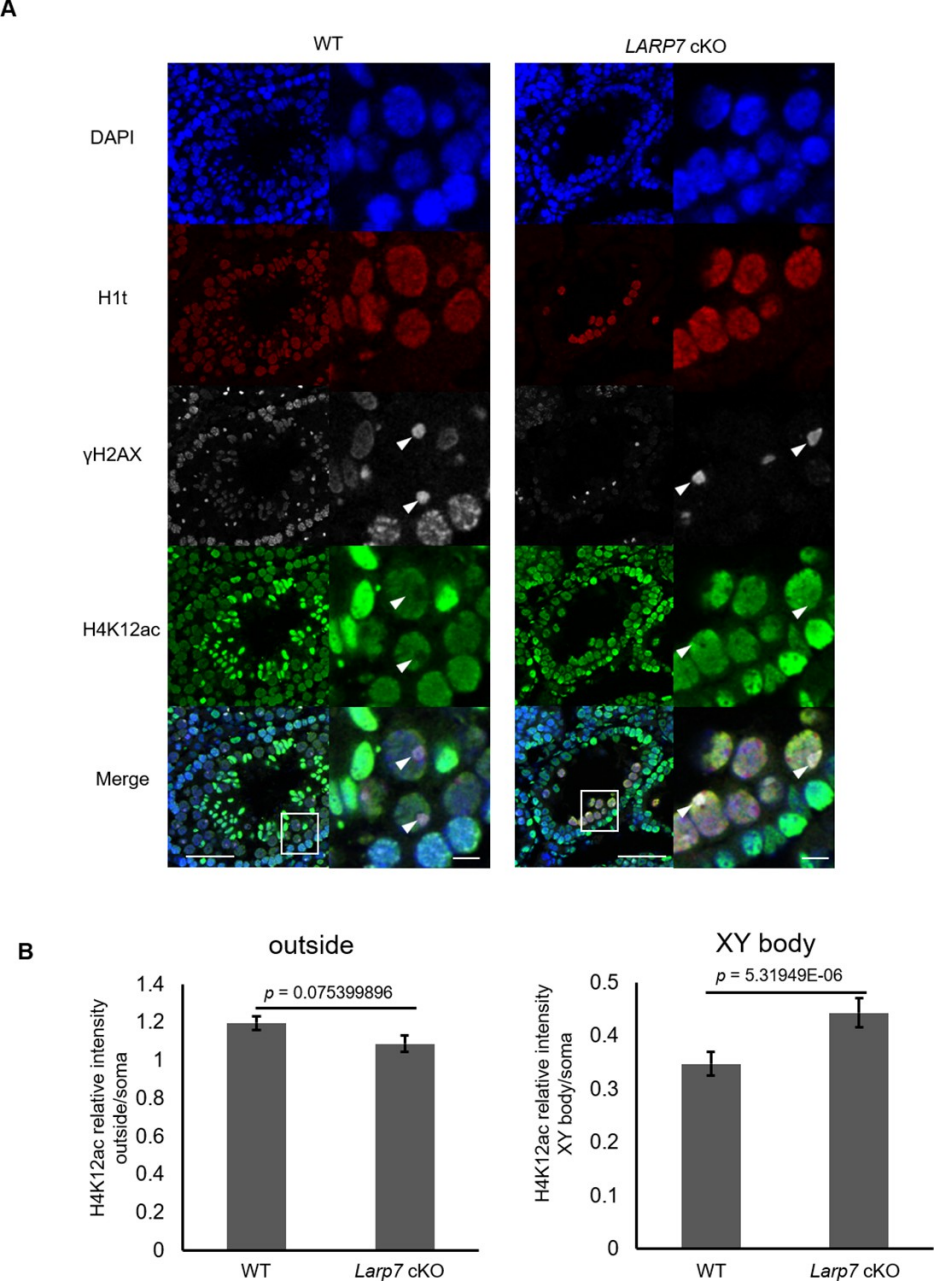
Abnormal localization of H4K12ac in the XY body of *Larp7* cKO mice. A. H1t (red), H4K12ac (green), and γH2AX (white) detected by immunostaining in the 5 weeks old testis section in wild type and *Larp7* cKO mice. Lower panels indicated magnified images corresponding to the rectangular areas in upper panels. Bars = 50 μm (upper panel) and 10 μm (lower panel). Arrowheads indicate the XY body. B. Quantification of relative intensity of H4K12ac outside the XY body in the nucleus (left) and in the XY body (right) in wild type and *Larp7* KO spermatocytes (Mean ± SE, WT: n = 150 cells, *Larp7* cKO: n = 147 cells from 3 animals).

**Fig 6:**
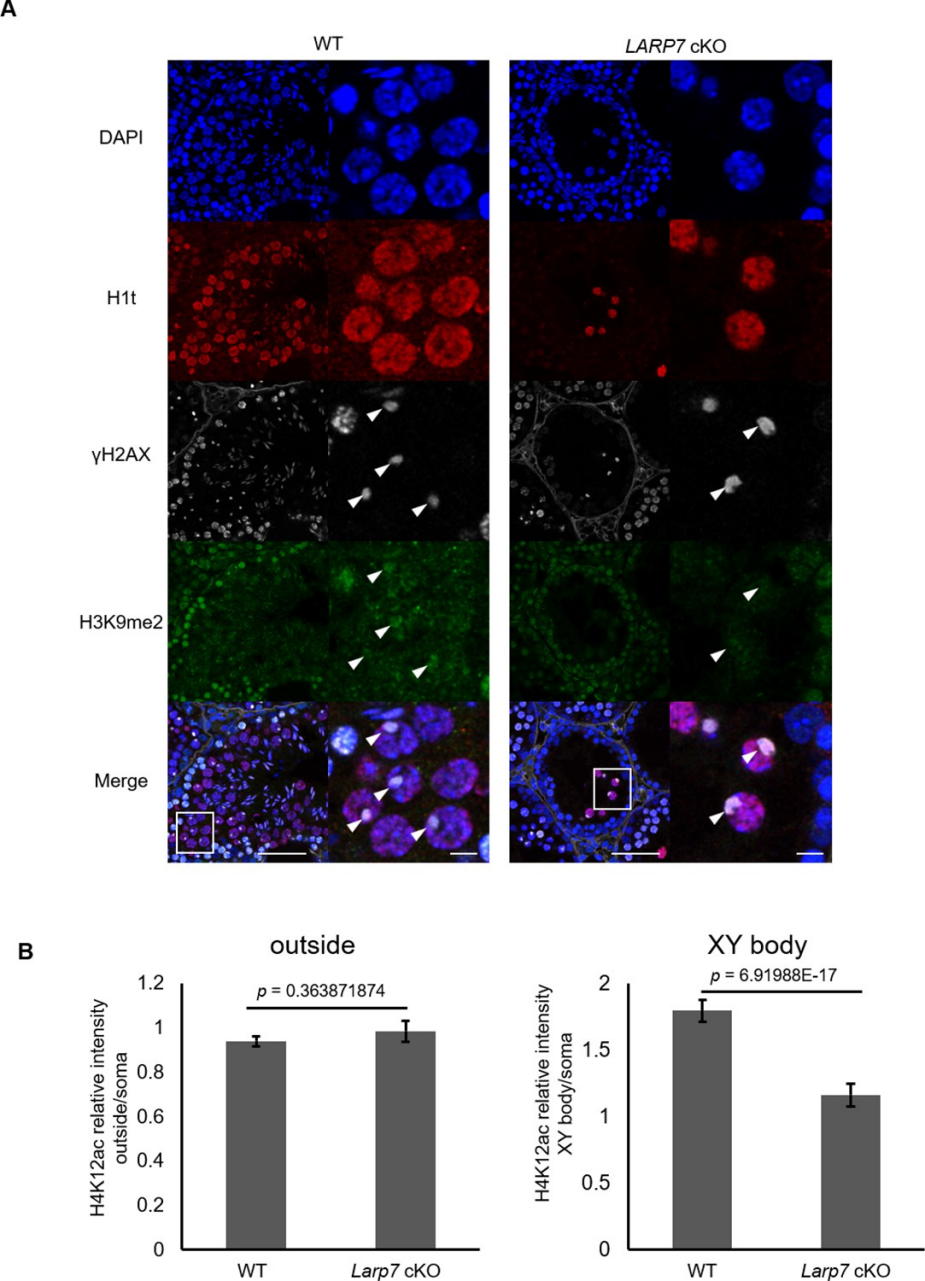
Abnormal localization of H3K9me2 in the XY body of *Larp7* cKO mice. A. H1t (red), H3K9me2 (green), and γH2AX (white) detected by immunostaining in the 5 weeks old testis section in wild type and *Larp7* cKO mice. Lower panels indicated magnified images corresponding to the rectangular areas in upper panels. Bars = 50 μm (upper panel) and 10 μm (lower panel). Arrowheads indicate the XY body. B. Quantification of relative intensity of H3K9me2 outside the XY body in the nucleus (left) and in the XY body (right) in wild type and *Larp7* KO spermatocytes (Mean ± SE, WT: n = 150 cells, *Larp7* cKO: n = 128 cells from 3 animals).

During MSCI, several histone modifications dynamically changed in the XY body, suggesting their involvement in MSCI [9, 34–36]. Therefore, we compared representative histone modifications including H3K4me2, H3K4me3, H3K9me2, H3K9me3, H3K27me3, H4K9ac, H4K12ac, and H4K16ac in the *Larp7* cKO spermatocyte with those in WT by immunostaining (Figs 5 and 6, S4 Fig). As a result, the signal intensity of H4K12ac in the XY body in H1t-expressing mid- to late pachytene spermatocytes was significantly higher in *Larp7* cKO spermatocytes than that in WT, while H4K12ac outside the XY body in nucleus was similarly observed in WT and *Larp7* cKO spermatocytes (Fig 5). The results suggest that overall H4K12ac in spermatocytes is not affected, but is preferentially enriched in the XY body by *Larp7* cKO, implying a role of LARP7 in accumulation of H4K12ac in the XY body. On the contrary, the signal intensity of H3K9me2 in the XY body in H1t-expressing mid- to late pachytene spermatocytes was significantly lower in *Larp7* cKO spermatocytes than that in WT, and H3K9me2 outside the XY body in nucleus was similarly observed in WT and *Larp7* cKO spermatocytes (Fig 6). Meanwhile, the tested other histone modifications were not significantly changed in the XY body of *Larp7* cKO spermatocytes (S4 Fig).

## Discussion

In this study, we provided new evidence that LARP7 is involved in the regulation of MSCI in the XY body in spermatocytes, which is likely regulated via exclusion and accumulation of H4K12ac and H3K9me2, respectively, in the XY body. In wild type testis, H4K12ac is excluded from the XY body of pachytene spermatocytes [9], which is consistent with the transcriptional repression of sex chromosomes as histone acetylation promotes gene transcription. We found that H4K12ac exclusion was diminished in the *Larp7* cKO spermatocyte (Fig 5), and it suggests that LARP7 plays a role on MSCI via exclusion of H4K12ac, though a possibility of H4K12ac accumulation in the *Larp7* cKO XY body as a consequence of MSCI failure cannot be excluded. The expression of histone deacetylase genes *Hdac7* and *Hdac8* are increased in *Larp7*-deficient spermatocytes (S2 Table), but H4K12ac levels outside the XY body in the spermatocyte nucleus are seemingly similar in *Larp7* cKO and in wild type spermatocytes, suggesting regulation of localized deacetylation activity, but not their expression by LARP7 in the XY body. Meanwhile, H3K9me2 is likely involved in the establishment of repressive chromatin in the XY body in wild type spermatocytes, and was diminished in *Larp7*-deficient XY body (Fig 6), suggesting that LARP7 controls H3K9me2.

Concerning LARP7 protein expression in postnatal testes, its expression temporarily becomes undetectable at P5 and P10, and resumes at P15 (Fig 1B). Although biological meanings of the temporal downregulation of LARP7 are currently unknown, during the absence of LARP7 in early spermatogonia, those cells might be released form CDKI-mediated cell cycle control, which is crucial in fetal germ cells [16], and resumed LARP7 protein in spermatocytes at P15 may newly establish its function in meiosis.

In *Larp7*-lacking pachytene spermatocytes, a few small foci of γH2AX are observed in autosomes, suggesting that DSBR is not completed in some autosomes. Similar abnormal DRBR was reported in spermatocytes of *Senataxin* (*Setx*) -deficient mice. SETX is a DNA/RNA helicase domain protein, and is considered as an essential component of the DNA damage response. It plays an important role in resolving R-loops (RNA:DNA hybrids that form over transcription pausing sites), transcription termination and maintaining genome stability [37]. SETX is localized in the XY body in spermatocytes, and its systemic knockout mice exhibit meiotic arrest and cell death at pachytene stage. As in the *Larp7*-deficient spermatocyte, γH2AX accumulation on sex chromosomes with its occasional small foci on autosomes are observed in *Setx* KO spermatocytes [37]. *Setx*-deficient spermatocytes also showed accumulation of H3K9ac and H4K16ac as well as failure of MSCI [38]. In addition, the study demonstrated that SETX assists in the deacetylation of histones to repress sex chromosome gene expression via association with chromodomain helicase DNA-binding protein 4 (CHD4), a component of the nucleosome remodeling complex (NuRD) [38]. Because SETX interacts with LARP7 in spermatocytes [17], it is likely that they functionally associate for MSCI.

In this study, we found meiotic arrest at late pachytene stage and devoid of spermatozoa in epididymis in germline-specific *Larp7* cKO mice, while the previous report has indicated remaining non-functional spermatozoa in the testes in addition to meiotic arrest at the spermatocyte stage [17]. The inconsistent phenotypes between the two *Larp7* cKO mice might be caused, at least in part, by difference in the Cre driver mice. *Stra8-Cre* mice were used in the previous report, which is expressed from 3 days after birth [39], while the expression of *Vasa-Cre* used in this study is first found in fetal testis at embryonic day 15 [21], and the earlier Cre expression from *Vasa-Cre* likely results in more severe phenotype. Detailed molecular mechanisms of MSCI by LARP7 need to await further analysis, which should shed new light on regulation of MSCI.

## Supporting information

S1 Fig The construction of germ cell-specific *Larp7* knockout mice. A. A schematic diagram illustrating the construction for conditional knockout of *Larp7* mice and the generation of germ cell-specific *Larp7*-null (*Larp7* cKO) mice by *Vasa*-*Cre* mediated recombination. Arrows indicate primer positions for genotyping PCR of floxed and wild type fragments. B. Representative electrophoresis images of genotyping for floxed / wild type *Larp7* (lane 1), wild type / homo (lane 2), and foxed / deleted *Larp7* (lane 3). C. RT-qPCR analyses of *Larp7* expression in spermatocytes from 5 weeks old wild type and *Larp7* cKO mice (Mean ± SE, n = 6). D. Western blot analysis of LARP7 in testes from 5 weeks old wild type and *Larp7* cKO mice. Left, representative images of membrane. Right, quantification of LARP7 signal intensity (Mean ± SE, n = 3). β-actin served as an internal normalized reference. E. Immunostaining of testis sections from 5 weeks old wild type and *Larp7* cKO mice. Left, representative images of the immunostaining for H1t (red), LARP7 (green), and γH2AX (white) with nuclei counterstained by DAPI (blue). Arrowheads indicate LARP7 signal and arrows indicate γH2AX signals. The magnified images of squared region are indicated in the right panels. Bars = 10 μm. Right, quantification of LARP7 signal intensity in the nucleus (Mean ± SE, 50 spermatocytes from 1 animal). LARP7 signal intensity of the mean value of the 50 somatic cells served as normalization.
(TIF)

S2 Fig Localization of MDC1 in *Larp7* cKO spermatocytes. Immunostaining of testis sections from 5 weeks old wild type and *Larp7* cKO mice for H1t (red), MDC1 (green), and γH2AX (white) with nuclei counterstained by DAPI (blue). Arrowheads indicate MDC1 signal and arrows indicate γH2AX signals. The magnified images of squared region are indicated in the right panels. Bars = 10 μm.
(TIF)

S3 Fig RNA-seq for *Larp7* cKO spermatocytes. A. qRT-PCR analysis of X chromosome genes (*Taf7l* and *Ccnb3*) and Y chromosome genes (*Ddx3y* and *Uba1y*) in spermatocytes from 5 weeks old wild type and *Larp7* cKO mice (Mean ± SE, n = 6). B. GO analysis of downregulated genes (logFC < -1, FDR < 0.05) in *Larp7* KO spermatocytes. C. GO analysis of upregulated genes (logFC > 1, FDR < 0.05) in *Larp7* KO spermatocytes.
(TIF)

S4 Fig Localization of histone modifications in the XY body of *Larp7* cKO mice. A-F. Representative images of H3K4me2 (green) (A), H3K4me3 (green) (B), H3K9me3 (green) (C), H3K27me3 (green) (D), H4K9ac (green) (E), H4K16ac (green) (F), H1t (red), and γH2AX (white) in 5 weeks old testis section in wild type and *Larp7* cKO (left), and quantification of relative intensity of each histone modification in the XY body (right, Mean ± SE, WT: n = 50 cells, *Larp7* cKO: n = 26 cells (A), n = 37 cells (B), n = 37 cells (C), n = 22 cells (D), n = 45 cells (E), n = 39 cells (F) from 1 animal). Bars = 10 μm.
(TIF)

S1 Table Primers used in this study. (XLSX)

S2 Table Changes of X and Y chromosome gene expression in RNA-seq. (XLSX)

S3 Table Upregulated genes and their GO analysis in Larp7 cKO spermatocyte. (XLSX)

S4 Table Downregulated genes and their GO analysis in Larp7 cKO spermatocyte. (XLSX)

S1 Raw image (PDF)

S2 Raw image (PDF)

S3 Raw image (PDF)
