# Supplementary figures and images for: LARP7 is required for sex chromosome silencing during meiosis in mice
Source: PLoS One. 2024 Dec 5;19(12):e0314329. doi: 10.1371/journal.pone.0314329 (PMC11620648; doi:10.1371/journal.pone.0314329)

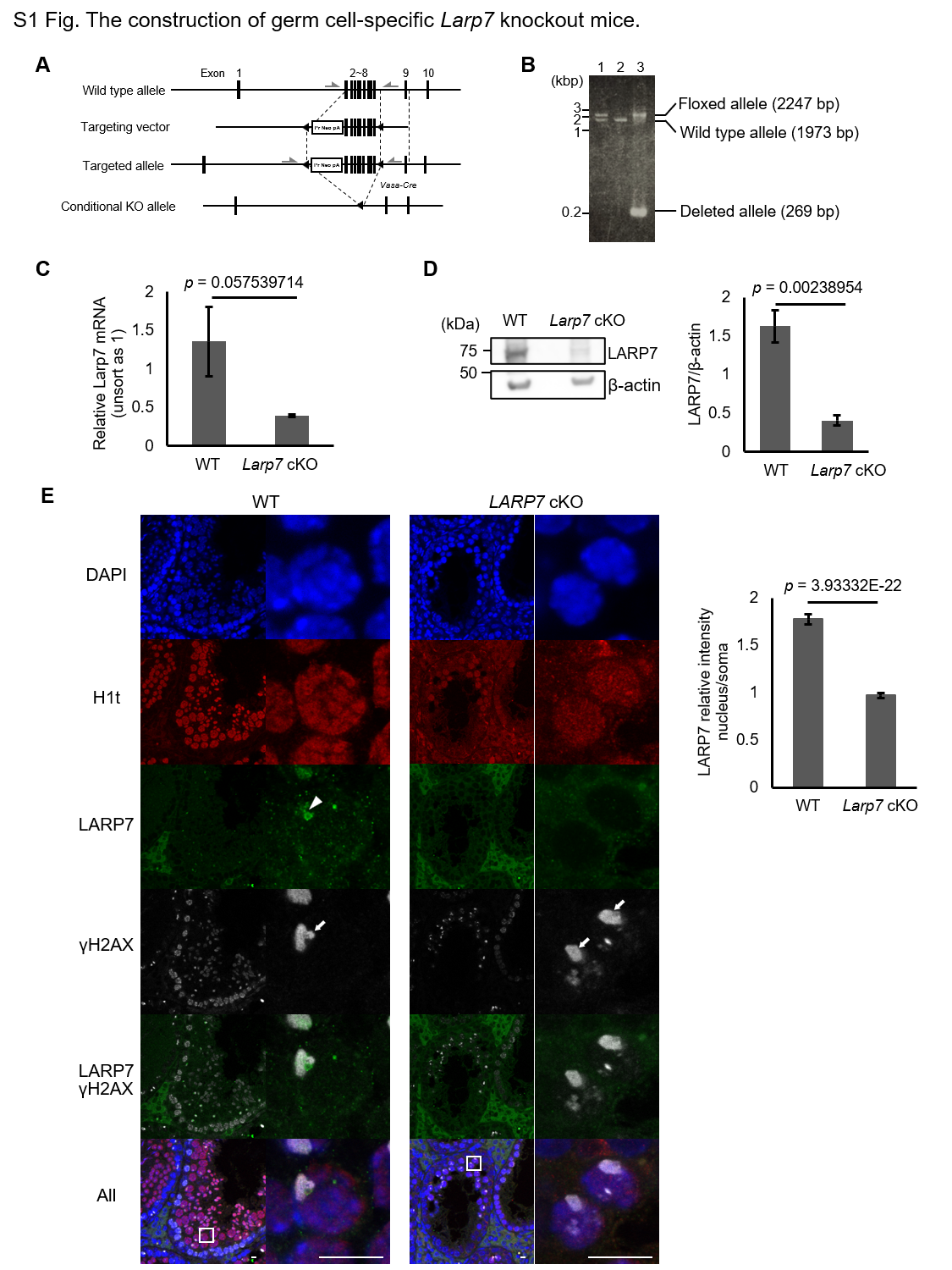

Supplement: S1 Fig — A. A schematic diagram illustrating the construction for conditional knockout of Larp7 mice and the generation of germ cell-specific Larp7-null (Larp7 cKO) mice by Vasa-Cre mediated recombination. Arrows indicate primer positions for genotyping PCR of floxed and wild type fragments. B. Representative electrophoresis images of genotyping for floxed / wild type Larp7 (lane 1), wild type / homo (lane 2), and foxed / deleted Larp7 (lane 3). C. RT-qPCR analyses of Larp7 expression in spermatocytes from 5 weeks old wild type and Larp7 cKO mice (Mean ± SE, n = 6). D. Western blot analysis of LARP7 in testes from 5 weeks old wild type and Larp7 cKO mice. Left, representative images of membrane. Right, quantification of LARP7 signal intensity (Mean ± SE, n = 3). β-actin served as an internal normalized reference. E. Immunostaining of testis sections from 5 weeks old wild type and Larp7 cKO mice. Left, representative images of the immunostaining for H1t (red), LARP7 (green), and γH2AX (white) with nuclei counterstained by DAPI (blue). Arrowheads indicate LARP7 signal and arrows indicate γH2AX signals. The magnified images of squared region are indicated in the right panels. Bars = 10 μm. Right, quantification of LARP7 signal intensity in the nucleus (Mean ± SE, 50 spermatocytes from 1 animal). LARP7 signal intensity of the mean value of the 50 somatic cells served as normalization. (TIF) [file pone.0314329.s001.tif]

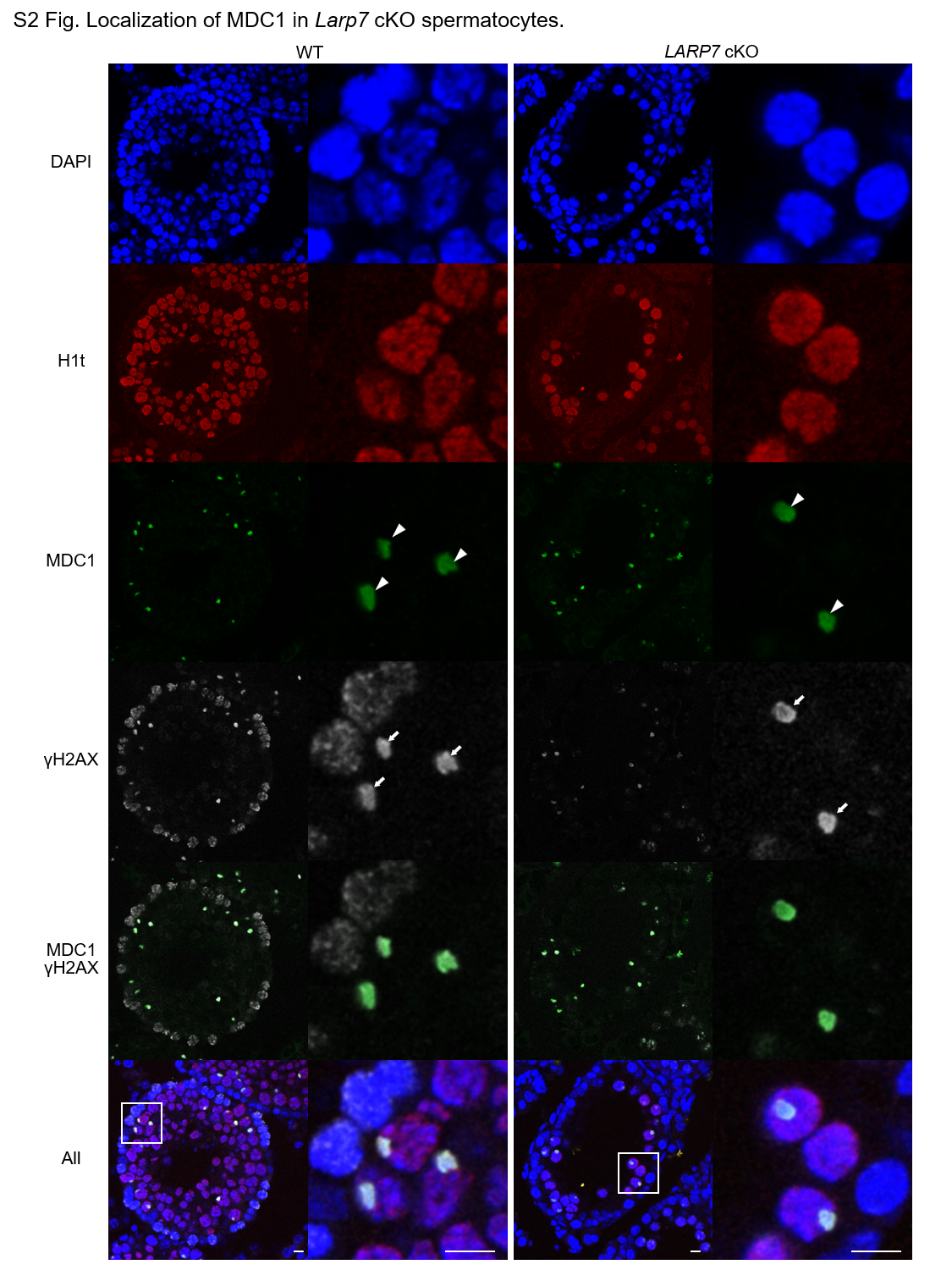

Supplement: S2 Fig — Immunostaining of testis sections from 5 weeks old wild type and Larp7 cKO mice for H1t (red), MDC1 (green), and γH2AX (white) with nuclei counterstained by DAPI (blue). Arrowheads indicate MDC1 signal and arrows indicate γH2AX signals. The magnified images of squared region are indicated in the right panels. Bars = 10 μm. (TIF) [file pone.0314329.s002.tif]

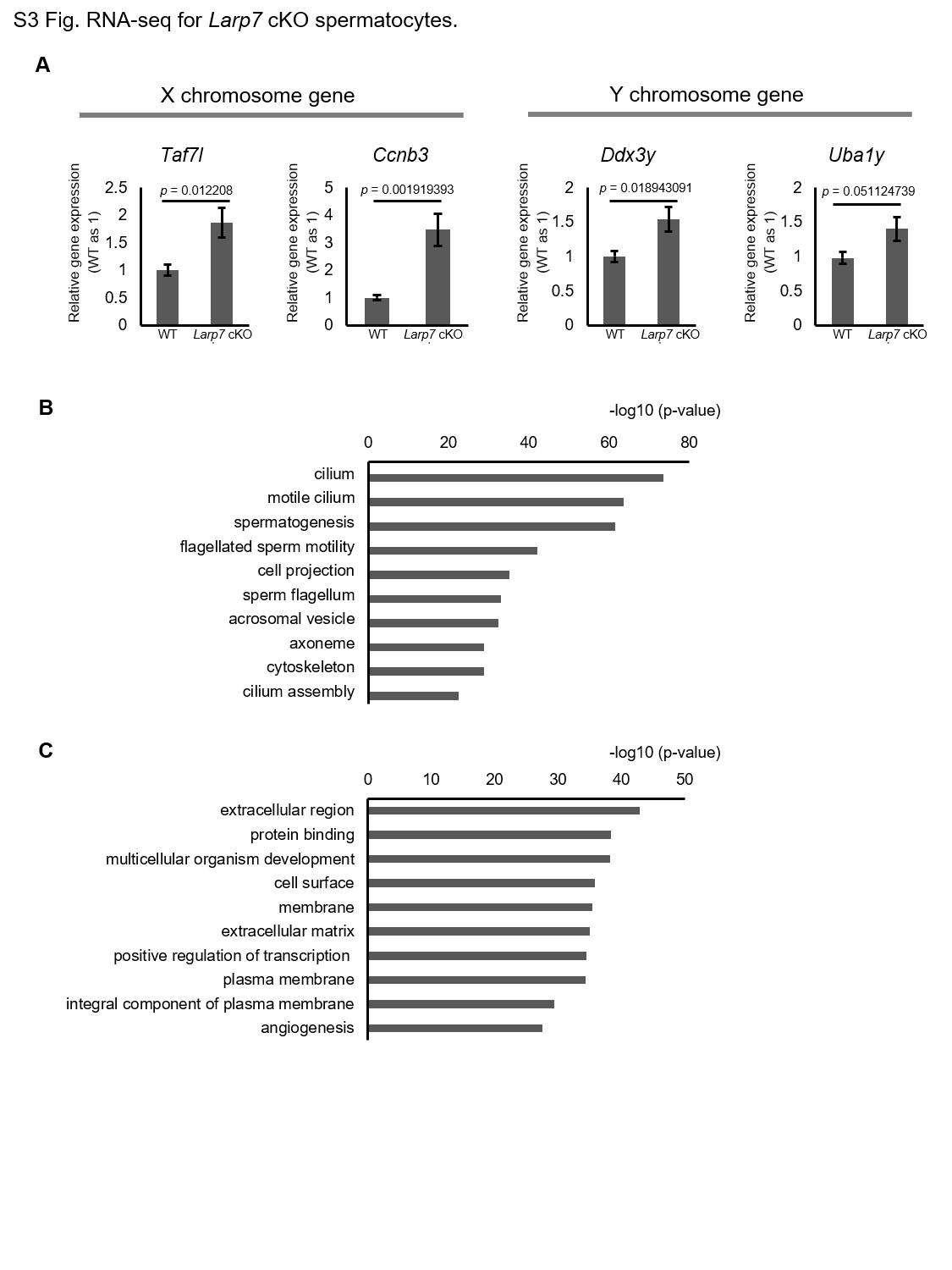

Supplement: S3 Fig — A. qRT-PCR analysis of X chromosome genes (Taf7l and Ccnb3) and Y chromosome genes (Ddx3y and Uba1y) in spermatocytes from 5 weeks old wild type and Larp7 cKO mice (Mean ± SE, n = 6). B. GO analysis of downregulated genes (logFC < -1, FDR < 0.05) in Larp7 KO spermatocytes. C. GO analysis of upregulated genes (logFC > 1, FDR < 0.05) in Larp7 KO spermatocytes. (TIF) [file pone.0314329.s003.tif]

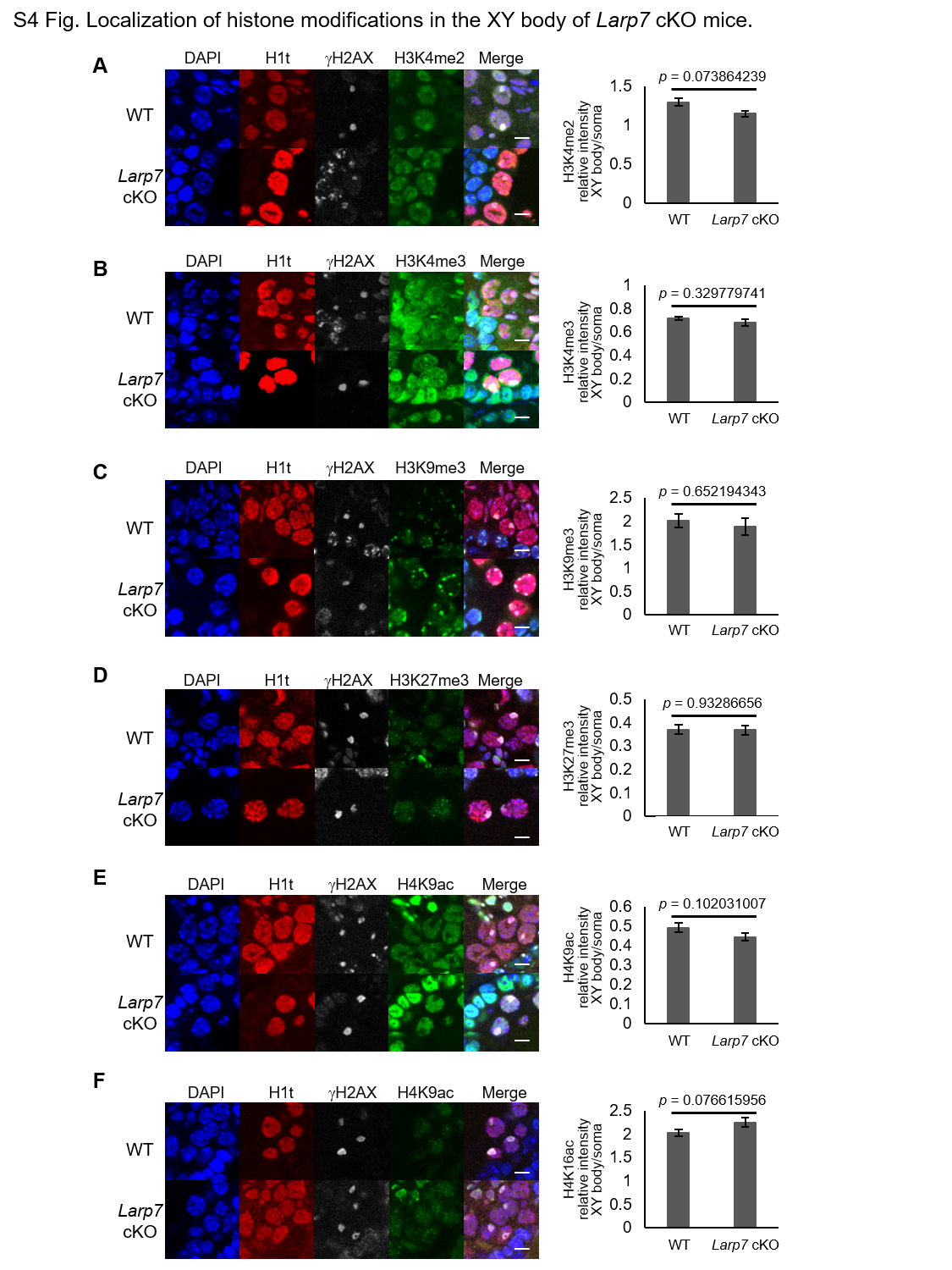

Supplement: S4 Fig — A-F. Representative images of H3K4me2 (green) (A), H3K4me3 (green) (B), H3K9me3 (green) (C), H3K27me3 (green) (D), H4K9ac (green) (E), H4K16ac (green) (F), H1t (red), and γH2AX (white) in 5 weeks old testis section in wild type and Larp7 cKO (left), and quantification of relative intensity of each histone modification in the XY body (right, Mean ± SE, WT: n = 50 cells, Larp7 cKO: n = 26 cells (A), n = 37 cells (B), n = 37 cells (C), n = 22 cells (D), n = 45 cells (E), n = 39 cells (F) from 1 animal). Bars = 10 μm. (TIF) [file pone.0314329.s004.tif]

1kb  
ladder

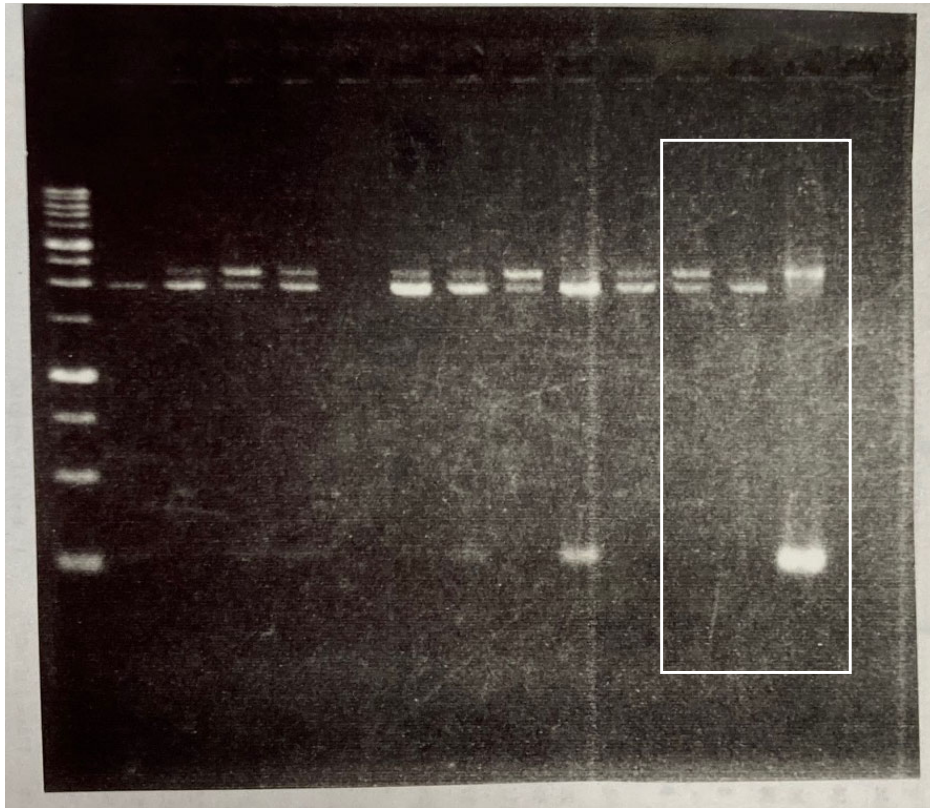

Supplement: S2 Raw image — (PDF) [file pone.0314329.s010.pdf]

Chemi

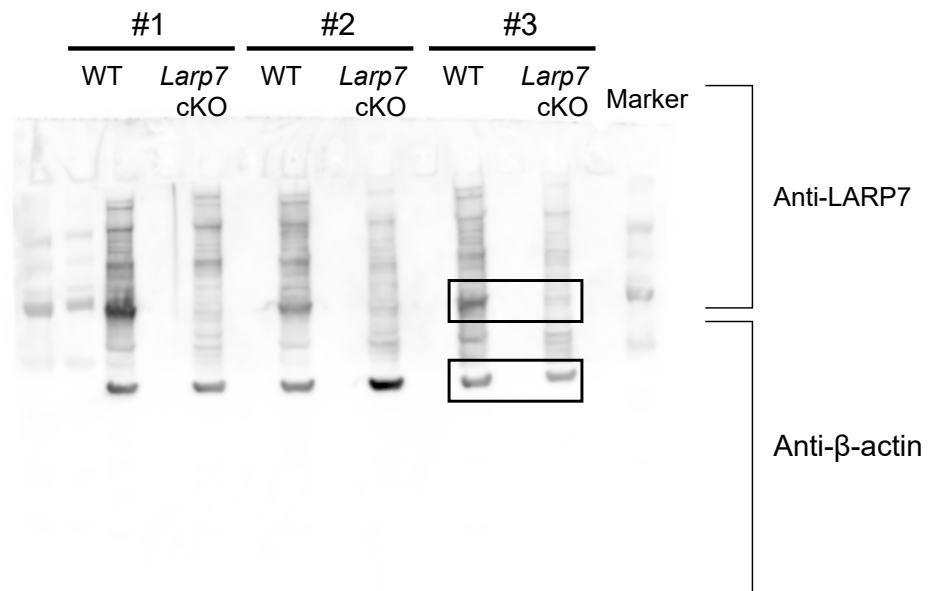

Chemi  
+  
Marker

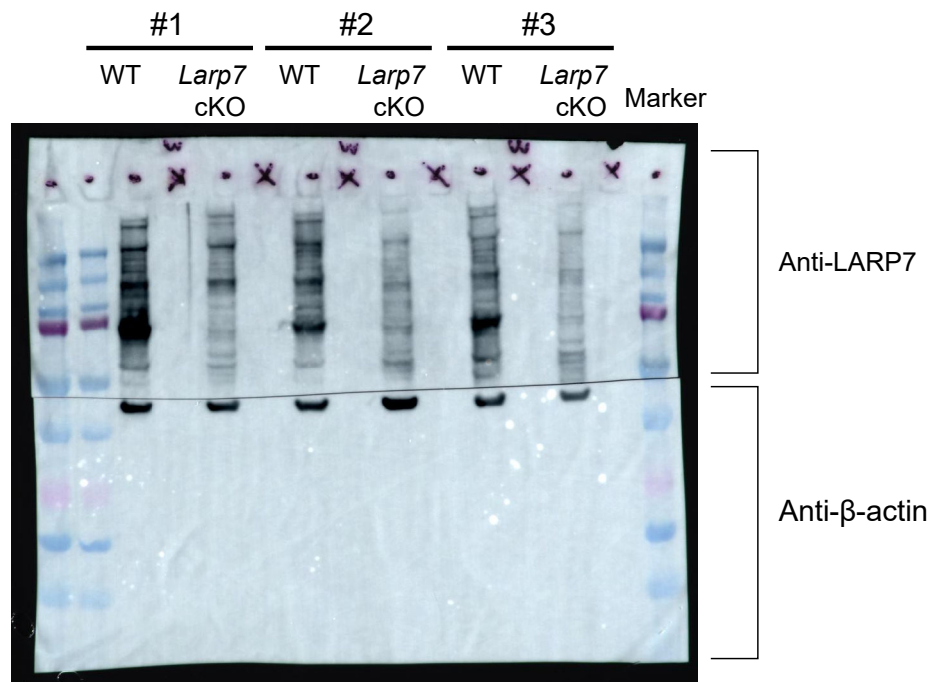

Supplement: S3 Raw image — (PDF) [file pone.0314329.s011.pdf]
