# Supplementary material for: LARP7 is required for sex chromosome silencing during meiosis in mice
Source: PLoS One. 2024 Dec 5;19(12):e0314329. doi: 10.1371/journal.pone.0314329 (PMC11620648; doi:10.1371/journal.pone.0314329)
Supplement: S1 Raw image — (PDF) [file pone.0314329.s009.pdf]

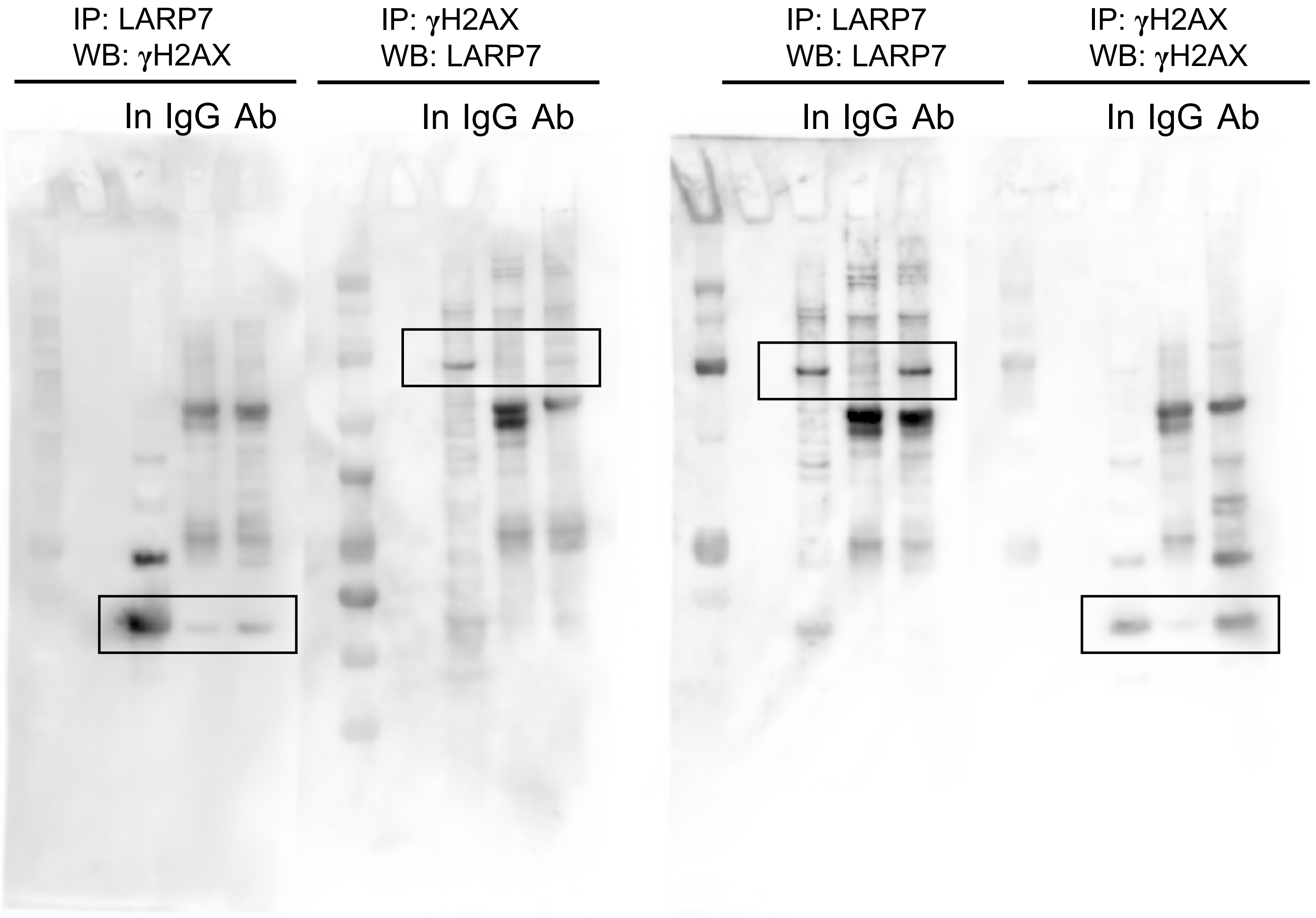
IP: LARP7
WB: γH2AX
In IgG Ab
IP: γH2AX
WB: LARP7
In IgG Ab
IP: LARP7
WB: LARP7
In IgG Ab
IP: γH2AX
WB: γH2AX
In IgG Ab
